# Detection of highly conductive surface electron states in topological crystalline insulators Pb_1−*x*_Sn_*x*_Se using laser terahertz radiation

**DOI:** 10.1038/srep11540

**Published:** 2015-06-22

**Authors:** S. G. Egorova, V. I. Chernichkin, L. I. Ryabova, E. P. Skipetrov, L. V. Yashina, S. N. Danilov, S. D. Ganichev, D. R. Khokhlov

**Affiliations:** 1 Physics Department, M.V. Lomonosov Moscow State University, Moscow 119991, Russia; 2 Chemistry Department, M.V. Lomonosov Moscow State University, Moscow 119991, Russia; 3 Faculty of Physics, University of Regensburg, Regensburg D-93040, Germany; 4 P.N. Lebedev Physical Institute of RAS, Moscow 119991, Russia

## Abstract

We suggest a method for detection of highly conductive surface electron states including topological ones. The method is based on measurements of the photoelectromagnetic effect using terahertz laser pulses. In contrast to conventional transport measurements, the method is not sensitive to the bulk conductivity. The method is demonstrated on an example of topological crystalline insulators Pb_1−*x*_Sn_*x*_Se. It is shown that highly conductive surface electron states are present in Pb_1−*x*_Sn_*x*_Se both in the inverse and direct electron energy spectrum.

Theoretical prediction of existence of gapless electron states on the surface of topological insulators (TI) stimulated extensive experimental studies directed to detection of these states^1^,^2^,^3^. By now, the main tools of this research are ARPES measurements that provided a convincing proof of existence of gapless electron states on the TI surface. Studies of electron transport via these states still remain a challenge. This electron transport is topologically protected against backscattering, and the carrier effective masses are very small due to the Dirac energy spectrum of the TI states. In most of the cases, however, this surface electron transport is shunted by conductivity via the bulk of a sample since most of the topological insulators are narrow-gap semiconductors with the Fermi level lying on the background of either conduction or valence band^4^,^5^,^6^. There are two approaches to overcome this difficulty. In the first approach, thin films or nanowires are used to increase the relative contribution of the surface electron transport. With this approach, manifestations of conductivity along Dirac surface states through magnetotransport measurements were reported for Bi_2_Se_3_ and SnTe nanowires and thin films^7^,^8^,^9^,^10^,^11^. The second approach uses optical excitation of surface electron states. Recently it has been demonstrated that the photogalvanics excited in Dirac Fermions^12^ provides an opto-electronic access to probe the electron transport in 3D TI even in dirty materials at the room temperature where conventional surface electron transport is hindered by the high carrier density in the bulk.

In this paper, we suggest a method for detection of highly conductive surface electron states including topological ones which is not sensitive to the presence of charge carriers in the bulk of a sample. This method uses the photoelectromagnetic effect induced by pulses of laser terahertz radiation.

The photoelectromagnetic (PEM) effect is appearance of a voltage drop *U*_*PEM*_ between sample contacts oriented perpendicular to the incident radiation direction and to the magnetic field (see insert in the Fig. 1).

It comes as a consequence of a diffusive flux of photoexcited electrons in magnetic field, in an analogy to the Hall effect, in which the electron flux is determined by the electric field applied. In the PEM effect, the magnitude and the sign of the signal is defined by the magnetic field and by the direction and the value of the diffusive carrier flux. The charge carrier flux may appear either as a consequence of photogeneration of excess carriers in the surface layer of a semiconductor, or due to heating of free charge carriers^13^. In our experiments, the energy of laser terahertz radiation quanta was much less than any characteristic parameters of the semiconductor energy spectrum, such as the energy gap, so no photogeneration of extra free carriers was possible. Therefore the effect was completely due to the charge carrier heating. In this sense, it is analogous to the Nernst effect in which the electron temperature gradient is induced by pulses of the terahertz radiation, while the lattice temperature remains unaffected.

The method is demonstrated by an example of topological crystalline insulators (TCI) Pb_1−*x*_Sn_*x*_Se. In this type of materials, crystalline symmetry replaces the role of time-reversal symmetry in ensuring topological protection. Pb_1−*x*_Sn_*x*_Se form a continuous range of solid solutions for all tin content values 0 < *x* < 1. The energy gap varies with *x* passing through zero at *x* = 0.15 at *T* = 0 K (Fig. 2)^14^. In the low tin content range *x* < 0.15, these semiconductors possess a positive gap value and are classified as trivial insulators. At *x* > 0.15, instead, the inverted energy spectrum is realized providing a background for appearance of non-trivial topologically protected surface electron states. Appearance of these topological helical Dirac-like electronic states was demonstrated by ARPES measurements^15^,^16^.

The typical PEM effect kinetics together with the laser pulse time profile is shown in the Fig. 1. The PEM effect signal basically repeats the shape of a laser pulse, no delay is observed. For all samples, the signal amplitude drops exponentially with increasing temperature *U*_*PEM*_ ∼ exp(−*T*/*T*_0_) (insert in the Fig. 1), *T*_0_ ≈ 10 K at the laser wavelength of 148 *μ*m and the highest power in the pulse of 30 kW. The effect is not observed anymore at *T* > 20 K. Further we will consider only data taken at *T* = 4.2 K when the signal amplitude is maximal.

Figure 2 shows dependence of the PEM effect amplitude on the magnetic field. The sign of the effect changes to the opposite when the magnetic field direction is reversed while its amplitude is not affected, i.e. the effect is odd in magnetic field. The effect amplitude first rises linearly in low fields *B* < 1.5 T, but then saturates and even has a tendency to drop in high fields. According to the theory of the PEM effect, this drop is due to the enhanced surface recombination^13^. The magnetic field corresponding to the effect maximum *B*_*m*_ satisfies the criterion for transition from classically weak to classically strong fields *μB*_*m*_ ∼ 1. The effect does not depend on the polarization of the incident terahertz radiation – linear or circular. In contrast to the PEM effect, photoconductivity was not observed in all of the samples studied. We also note that the selected experimental geometry excludes the magneto-gyrotropic effect, another magneto-optical phenomenon which by symmetry arguments can be induced in the TI surface states for the in-plane magnetic field only^17^.

The PEM effect was detected not only in samples possessing the inverse energy spectrum with *x* > 0.15, but in samples with the direct spectrum with *x* < 0.15 as well. The sign, amplitude and characteristic features of the effect do not differ substantially from those observed for samples with *x* > 0.15. Moreover, in samples with *x* = 0.09 and 0.125 with the highest electron mobility, oscillations of the PEM effect in magnetic field were observed. These oscillations are most pronounced in the sample with *x* = 0.09 (Fig. 3).

The oscillations are equidistant in reverse magnetic field, and their period does not depend on the energy of the incident radiation quanta. It means that the origin of the oscillations may not be related to the magnetophonon effect^13^. Magnetoresistance Shubnikov – de Haas oscillations have been observed in the same magnetic field orientation, and their period is close to the PEM effect oscillation period (Fig. 3). This result means that the observed PEM effect is related not only to surface, but to the bulk energy states of the semiconductor as well since the magnetic field was directed parallel to the sample surface, and Landau quantization of surface conductive states was not possible.

It is important to note that the sign of the effect corresponds to the net diffusion of charge carriers from the surface to the sample bulk. The origin of the “classical” PEM effect comes from spatial separation of optically excited electrons and holes which diffuse from the sample surface in magnetic field^13^. Since photogeneration of both free electrons and holes is necessary, this effect does require that the optical photon energy exceeds the semiconductor bandgap. In our case, however, the radiation quantum energy is much less than the gap *E*_*g*_ and even the distance (*E*_*F*_ − *E*_*C*_) between the Fermi level and the conduction band edge. Therefore generation of electron-hole pairs is impossible, and the diffusive electron flux may be due only to heating of the electron gas by the terahertz laser radiation without changing its concentration. It is reasonable to assume that the degree of this heating is given by the temperature *T*_0_ ≈ 10 K.

The origin of appearance of the diffusive electron flux upon heating by terahertz radiation comes from the difference in charge carrier mobility of excited electrons nearby the sample surface and the electron mobility in the bulk of a sample. The excited electrons diffuse to the sample bulk while losing their energy. This leads, in turn, to counter diffusion of “cold” electrons from the bulk to the surface to provide the electric neutrality. In course of this process, the electron mobility gradient gives rise to a net flux of electrons^13^.

In homogeneous samples, the direction of the net electron flux is defined by the energy dependence of the free charge carrier mobility. If the mobility of excited charge carriers at the surface is higher than in the bulk, the net electron flux is directed from the sample surface to its bulk, in the opposite case, instead, it is directed from the bulk to the surface. In contrast to the Hall effect, the sign of the PEM effect depends only on this direction of the free charge carrier flux and does not depend on the charge carrier type – *n* or *p*.

The main scattering mechanism that defines the mobility temperatures dependence at high temperatures *T* > 30 K in Pb_1−*x*_Sn_*x*_Se and the congener compound Pb_1−*x*_Sn_*x*_Te is the phonon scattering. It provides a drop in the electron mobility with increasing electron temperature. In relation to the PEM effect, this drop gives rise to a net electron flux directed from the bulk to the surface with the respective *U*_*PEM*_ sign. It was observed previously in Pb_1−*x*_Sn_*x*_Te samples with low free electron concentration^18^.

At the low temperatures *T* < 20 K, instead, a temperature independent scattering mechanism becomes predominant since the electron concentration does not change and the resistivity saturates. It may be either the neutral impurity scattering or the strongly screened charged impurity scattering. In both cases, the number of scattering particles does not change with temperature. As shown in our experiment, the free electrons are heated by 10 K by the laser radiation. The free electron mobility either does not change or only slightly drops at 4.2 K < *T* < 15 K in Pb_1−*x*_Sn_*x*_Se^19^. Therefore it is reasonable to assume that the mobility either remains constant or slightly drops with increasing energy, too. In such a case, heating of electrons may lead only to a drop in their mobility and not to its rising. Therefore heating of electrons by terahertz laser pulses may provide only the net electron flux directed from the sample bulk to its surface. Experimentally, the opposite PEM effect sign is observed.

The reasons for such an unexpected behavior could arise from two factors. First, there is a possibility that the net electron flux to the bulk could be due not to a change in free electron mobility, but to a change in the free electron concentration. This, in turn, may come out as a result of excitation of surface local electron states with very low binding energies. However a very high dielectric permittivity of the material (ϵ ∼ 300^20^) prevents band bending on the semiconductor surface, as well as formation of surface local hydrogen-like electron states. Therefore this possibility is ruled out. The second option is appearance of highly conductive surface electron states with enhanced free electron mobility compared to the bulk. Our experimental data unambiguously demonstrate presence of such states in Pb_1−*x*_Sn_*x*_Se.

If the PEM effect would be observed only in samples with the inverted energy spectrum with *x* > 0.15, it would be reasonable to ascribe it to topological surface states of the TCI. However the effect is observed in samples with *x* < 0.15 as well. It means that the origin of the highly conductive surface electron states responsible for the effect is different. Appearance of highly conductive surface electron states both in the direct and inverse energy spectrum was predicted theoretically for TCI Pb_1−*x*_Sn_*x*_Se and Pb_1−*x*_Sn_*x*_Te^21^. The processes arising on the surface of potentially TCI systems even in the absence of a topological phase may provide an enhanced mobility of electrons in the highly conductive surface states. Presence of these states may shield the influence of a topological surface layer, if present.

The PEM effect amplitude changed by no more than a factor of 3–4 in different samples. This variation could result from slight difference in the experiment geometry for different samples. Another possible origin for such a variation could come from the scatter of the bulk electron mobility in different samples. On the other hand, the free electron concentration varied by more than two orders of magnitude. It means that the suggested method for detection of highly conductive surface electron states is not sensitive to the free electron concentration in the bulk. It is possible that the method may be applied for other TCI and TI systems as well. A criterion for involvement of topological surface states in the effect observed would be presence of the effect in the TCI or TI state of a system with the inverse energy spectrum and its absence in the situation with the direct spectrum.

In conclusion, we have suggested a method for detection of highly conductive surface electron states with enhanced mobility using the photoelectromagnetic effect induced by terahertz laser pulses. It is experimentally demonstrated that the method is not sensitive to the bulk free carrier concentration. The observed effect provides a selective photo-electric access to the electron transport in TI and, similarly to the photogalvanic spectroscopy in semiconductor quantum well systems^22^, can be applied to study tiny details of the band structure and carrier dynamics. It is shown that highly conductive surface electron states with enhanced mobility are present in the topological crystalline insulators Pb_1−*x*_Sn_*x*_Se both in the direct and inverse energy spectrum. Therefore it is demonstrated that even if existence of the topological states is confirmed by ARPES experiments (as it is in Pb_1−*x*_Sn_*x*_Se alloys with the inverted spectrum at *x* > 0.15^15^), observation of highly conductive surface electron states in electronic transport does not necessarily mean that these states are topological. There may exist other highly conductive surface electron states that hinder the topological states in transport measurements. In principle, these “hindering” non-topological surface electron states may reveal themselves not only in the PEM effect, but in other transport experiments as well.

## Methods

**Figure 2:**
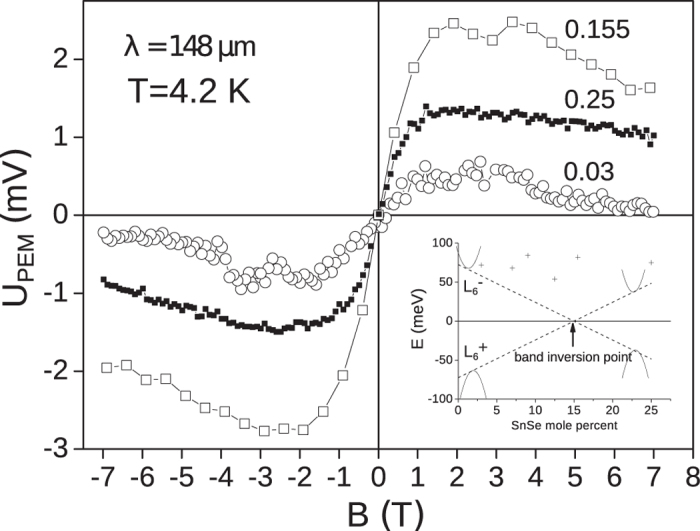
Magnetic field dependence of the PEM effect amplitude. Figures near the curves correspond to the composition *x* of Pb_1−*x*_Sn_*x*_Se samples. The sample temperature is 4.2 K, the laser wavelength is 148 *μ*m, the maximal power in a pulse is 30 kW. The insert shows the composition dependence of the conduction and valence band positions relative to the middle of the bandgap at *T* = 0 K together with the calculated positions of the Fermi level in the samples studied.

The samples under study were single crystals of Pb_1−*x*_Sn_*x*_Se with the 〈100〉 surface orientation grown by the Bridgman method^23^. The main sample parameters are given in the Table 1. All samples possessed the n-type conductivity. The sample composition *x* was determined using the X-ray fluorescence analysis. The resistivity *ρ*, free electron concentration *n* and mobility of free electrons *μ* were provided by measurements in the Hall bar geometry at temperatures 4.2—100 K. In this temperature range, the free electron concentration did not change for all samples, while the resistivity dropped with lowering temperature from 100 to about 30 K with further saturation down to 4.2 K. This temperature behavior of galvanomagnetic parameters is typical for nominally undoped Pb_1−*x*_Sn_*x*_Se alloys^14^. The direct bandgap *E*_*g*_ in the samples studied was calculated assuming that the bandgap in PbSe is *E*_*g*_ = 145 meV at the liquid-helium temperature, and it decreases with the rate of *dE*_*g*_/*dx* ≈ −9.7 meV/mol.% in Pb_1−*x*_Sn_*x*_Se alloys^14^. The calculation of the Fermi level position relative to the conduction band edge *E*_*F*_ − *E*_*C*_ was done using the six-band Dimmock model^24^ with the energy spectrum parameters given in the paper^25^. The respective position of the actual bands and the Fermi level in the samples studied are shown in the insert in Fig. 2.

100 ns – long pulses of an optically pumped NH_3_ terahertz laser^26^,^27^ with the wavelengths of 90, 148 and 280 *μ*m and the power in a pulse up to 30 kW were used to excite the PEM effect. The incident radiation power was monitored by a fast photon drag reference detector^28^. The beam has an almost Gaussian profile, which is measured by pyroelectric camera^29^. The magnetic field up to 7 T was applied to induce the PEM effect.

## Additional Information

**How to cite this article**: Egorova, S. G. *et al*. Detection of highly conductive surface electron states in topological crystalline insulators Pb_1–x_Sn_x_Se using laser terahertz radiation. *Sci. Rep*. **5**, 11540; doi: 10.1038/srep11540 (2015).

## Figures and Tables

**Figure 1:**
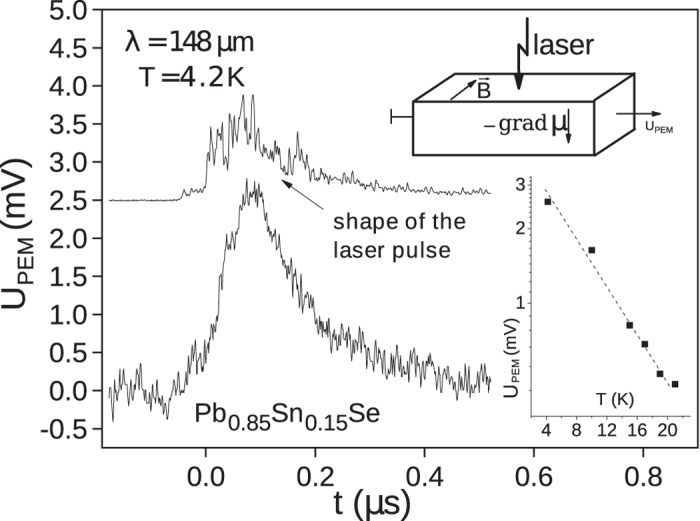
A typical kinetics of the photoelectromagnetic effect. The upper curve shows the respective laser pulse time profile. The inserts demonstrate the measurement geometry and the temperature dependence of the PEM signal.

**Figure 3:**
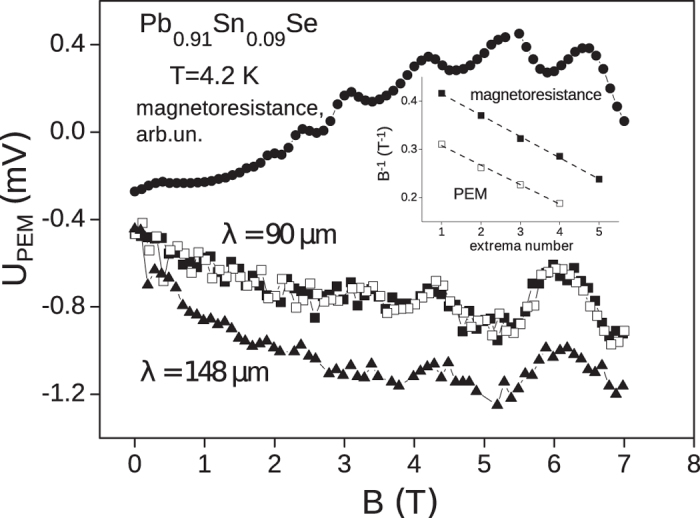
Magnetic field dependence of the PEM effect amplitude for the sample Pb_0.91_Sn_0.09_Se. Data in the figure correspond to the laser wavelengths 148 *μ*m (triangles) and 90 *μ*m (squares). Open square points are taken for increasing field, full squares correspond to decreasing field. The solid circles stand for the magnetoresistance measurements performed in the same geometry. The insert shows the extrema position in the reverse magnetic field as a function of the extremum number (points). The lines correspond to the rms interpolation of the data, the periods Δ(1/*B*) are 0.080 and 0.088 for the *U*_*PEM*_ and the magnetoresistance, correspondingly.

**Table 1:** Parameters of investigated Pb_1−*x*_Sn_*x*_Se samples at *T*  = 4.2 K.

*x*	*n* (cm^−3^)	*ρ* (Ω ⋅ cm)	*μ* (cm^2^V^−1^s^−1^)	*E*_*g*_ (meV)	*E*_*F*_ − *E*_*C*_ (meV)	(m^*^_100_(*E*_*g*_))/(*m*_0_)
0.03	1.0 × 10^17^	3.1 × 10^−3^	2.0 × 10^4^	116	7.9	0.033
0.07	3.5 × 10^17^	1.8 × 10^−3^	9.8 × 10^3^	77	20.9	0.031
0.09	1.0 × 10^18^	3.0 × 10^−5^	2.0 × 10^5^	58	39.8	0.039
0.125	3.0 × 10^17^	2.0 × 10^−4^	1.0 × 10^5^	24	29.8	0.025
0.155	1.1 × 10^18^	2.5 × 10^−4^	2.3 × 10^4^	−5	56.1	0.038
0.25	4.0 × 10^17^	1.8 × 10^−3^	8.7 × 10^3^	−97	10.6	0.035
0.37	3.2 × 10^19^	2.5 × 10^−3^	80	−211	18.4	0.130
